# Draft Genome Sequence of Moraxella bovoculi Strain KZ-1, Isolated from Cattle in North Kazakhstan

**DOI:** 10.1128/MRA.00670-20

**Published:** 2020-07-23

**Authors:** Marat Kuibagarov, Asylulan Amirgazin, Gilles Vergnaud, Alexandr Shustov, Anara Ryskeldina, Yerlan Ramankulov, Alexandr Shevtsov

**Affiliations:** a National Center for Biotechnology, Nur-Sultan, Kazakhstan; b Institute for Integrative Biology of the Cell (I2BC), Université Paris-Saclay, Gif‐sur‐Yvette, France; University of Maryland School of Medicine

## Abstract

Moraxella bovoculi strain KZ-1 was isolated from cattle that had symptoms of infectious bovine keratoconjunctivitis (IBK) in northern Kazakhstan. Here, we report the draft genome sequence of this strain.

## ANNOUNCEMENT

Infectious bovine keratoconjunctivitis (IBK) is a cattle disease that recently has become an emerging problem for veterinary medicine in Kazakhstan. As was reported by local veterinary doctors, IBK cases with the specific pathology of corneal ulceration were observed first in herds of imported Angus cattle. The IBK cases for which etiology was laboratory confirmed were caused by *Mоraxella* sp., and the etiological agent that was isolated was Moraxella bovis (1). Thus, it was postulated that IBK caused by *Mоraxella* sp. infection has been introduced into the territory of Kazakhstan with imported cattle (2). However, presently in the country, IBK has been recorded in all cattle breeds, including locally developed breeds, e.g., the Kazakh white-headed and Aulekol breeds. Beginning in 2002, a different pathogenic species, Moraxella bovoculi, began to be isolated from calves with IBK. Thus, at present, at least two *Moraxella* species participate in transmission and cause the pathogenic process (3). There is evidence that, depending on the main affected organs (the nasopharynx or eyes in animals with IBK), isolates of Moraxella bovoculi differ in genome structure, gene content, DNA polymorphism diversity, and DNA studies, allowing placement of the various isolates into separate phylogenetic groups (4).

Moraxella bovoculi strain KZ-1 was isolated by collecting samples of the mucous exudates from the eyes of diseased cows (Kazakh white-headed breed), with subsequent culturing on Colombian agar containing 5% sheep blood. The initial samples were collected in the North Kazakhstan region of Kazakhstan. Determination of the isolate was accomplished by analyzing the 16S rRNA gene fragment. Prior to isolation of genomic DNA, isolated single colonies were subcultured in brain heart infusion (BHI) broth at 37°C with stirring (150 rpm/min). Genomic DNA was isolated from the cultured bacterial strain using the QIAamp DNA minikit (Qiagen). DNA libraries were prepared using the Nextera XT DNA library prep kit (Illumina, San Diego, CA). Sequencing was performed on the MiSeq system using the MiSeq reagent kit v.3 (600 cycles, 2 × 300 bp). As a result, 1,218,680 reads were generated. Trimming of the noninformative sequence ends to Q30 values was performed using Geneious Prime (v.2019.2) and the BBDuk trimmer plugin v.1.0 using the default parameters. The reads were assembled *de novo* with SKESA v.2.3.0 with default parameters (5). The contigs produced were verified by BLAST v.2.9.0 against the NCBI GenBank nucleotide database of nonredundant sequences (6). The assembly quality was evaluated using QUAST v.5.0.2 (7). As a result, 27 contigs and a genomic sequence of 2,105,166 bp were produced. The average sequencing depth was 150×, the *N*_50_ value was 1,628,242 bp, and the genome GC content was 45.62%. Genome annotation was performed using the NCBI Prokaryotic Genome Annotation Pipeline (PGAP) v. 4.11 with default parameters (8, 9). As a result, 2,043 genes were found, including 1,989 coding DNA sequences (CDSs) (total) and 54 RNAs (7 rRNAs, 43 tRNAs, and 4 noncoding RNAs [ncRNAs]).

### Data availability.

This whole genome shotgun project has been deposited in DDBJ/ENA/GenBank under the accession no. JAAIVK000000000. The version described in this report is the first version, JAAIVK010000000. The raw data from BioProject accession no. PRJNA604664 were submitted to the NCBI SRA under the accession no. SRR11012145.

## References

[1] Alexander D 2010 Infectious bovine keratoconjunctivitis: a review of cases in clinical practice. Vet Clin North Am Food Anim Pract 26:487–503. doi:10.1016/j.cvfa.2010.09.006. 21056797

[2] Ivanov NP, AM Namet, Sattarova RS, Shynybaev KM, Akmyrzaev NZ, Issakulova BZ, Bakieva FA 2019 Moraxellosis in catches of different breeds of meat direction of productivity. Natl Acad Sci Repub Kazakh 2:78–82. doi:10.32014/2019.2224-526X.20.

[3] Angelos JA, Spinks PQ, Ball LM, George LW 2007 Moraxella bovoculi sp nov, isolated from calves with infectious bovine keratoconjunctivitis. Int J Syst Evol Microbiol 57:789–795. doi:10.1099/ijs.0.64333-0. 17392208

[4] Dickey AM, Loy JD, Bono JL, Smith TPL, Apley MD, Lubbers BV, DeDonder KD, Capik SF, Larson RL, White BJ, Blom J, Chitko-McKown CG, Clawson ML 2016 Large genomic differences between Moraxella bovoculi isolates acquired from the eyes of cattle with infectious bovine keratoconjunctivitis versus the deep nasopharynx of asymptomatic cattle. Vet Res 47:31. doi:10.1186/s13567-016-0316-2. 26872821 PMC4752781

[5] Souvorov A, Agarwala R, Lipman DJ 2018 SKESA: strategic k-mer extension for scrupulous assemblies. Genome Biol 19:153. doi:10.1186/s13059-018-1540-z. 30286803 PMC6172800

[6] Altschul SF, Gish W, Miller W, Myers EW, Lipman DJ 1990 Basic local alignment search tool. J Mol Biol 215:403–410. doi:10.1016/S0022-2836(05)80360-2. 2231712

[7] Gurevich A, Saveliev V, Vyahhi N, Tesler G 2013 QUAST: quality assessment tool for genome assemblies. Bioinformatics 29:1072–1075. doi:10.1093/bioinformatics/btt086. 23422339 PMC3624806

[8] Zhao Y, Wu J, Yang J, Sun S, Xiao J, Yu J 2012 PGAP: pan-genomes analysis pipeline. Bioinformatics 28:416–418. doi:10.1093/bioinformatics/btr655. 22130594 PMC3268234

[9] Tatusova T, DiCuccio M, Badretdin A, Chetvernin V, Nawrocki EP, Zaslavsky L, Lomsadze A, Pruitt KD, Borodovsky M, Ostell J 2016 NCBI Prokaryotic Genome Annotation Pipeline. Nucleic Acids Res 44:6614–6624. doi:10.1093/nar/gkw569. 27342282 PMC5001611

